# Influence of Visual Color Cues on Saltiness Expectation, Sensory Liking, and Emotions: A Soy Sauce Model Study

**DOI:** 10.3390/foods15010159

**Published:** 2026-01-03

**Authors:** Peerapong Wongthahan, Amporn Sae-Eaw, Witoon Prinyawiwatkul

**Affiliations:** 1 Department of Food Technology, Faculty of Technology, Khon Kaen University, Khon Kaen 40002, Thailand; peerwo@kku.ac.th; 2 School of Nutrition and Food Sciences, Louisiana State University Agricultural Center, Baton Rouge, LA 70803, USA; wprinya@lsu.edu

**Keywords:** visual color cues, soy sauce, saltiness, consumer perception, expectation, liking, emotion

## Abstract

Food color can greatly impact consumer perception. It can shape flavor expectations and influence emotions. This research evaluated how visual color cues affect perceived saltiness, sensory liking, and emotional responses to soy sauce. The study used four samples with the same salt concentrations (12% NaCl *w*/*v*). The color was varied in intensities, including light (LS), control (CS), moderate (MS), and high (HS). There was a total of 100 consumers to evaluate the samples. The results showed that MS received the highest liking scores for color (6.17) and saltiness (6.30). LS achieved the lowest scores at 3.98 for color and 5.78 for saltiness. Color intensity had a significant correlation with the expectation of saltiness. Correspondence analysis revealed that MS was most frequently associated with positive emotions such as “interested” (36%), whereas LS evoked negative emotions, including “disgusted,” “bored,” and “worried.” These findings confirm that darker colors enhance perceived taste intensity and positive affect. The use of color cues may therefore be a simple strategy to design low-sodium soy sauce formulations without reducing consumer acceptance while potentially supporting sodium reduction initiatives aimed at improving public health outcomes.

## 1. Introduction

Consumers greatly depend on visual cues to understand their surrounding environment. This process refers to visual perception and has an important role in how food quality is judged before tasting [1]. Food color contains information about freshness, flavor intensity, and product characteristics. Thus, it impacts consumers’ expectations, sensory evaluation, and purchase decisions [2]. However, food color is interpreted differently in different products. For instance, red is often linked with good taste and auspiciousness in Chinese cuisine [3]. Even the same color can cause contradictory meanings. A dark hue can suggest richness in chocolate but mean overcooking in meat [4,5].

There is a strong cross modal connection between color and flavor expectation [6]. Consumers show a tendency to link specific hues with basic tastes, such as bitterness with black, saltiness with white, sourness with green, and sweetness with pink [7]. Adjusting color in foods can systematically change taste perception. For example, increasing red or green can increase the perception of sweetness or sourness [8]. In addition to basic sensory perception, color also works as a marketing cue. It differentiates products and shapes consumer expectations. Research in sensory marketing has shown that consumers associate specific colors with their emotions. This can cause them to make impulsive purchases or increase purchase intent for familiar products [9]. Such evidence indicates that food color not only affects perceived flavor, odor, and taste but also impacts consumers’ overall food choice and purchase behavior [10].

Seasoning, dressing, and condiments are a major part of cuisine across cultures all around the world and are used to enhance the flavor of food. Among them, soy sauce, which is made from fermented soybean (*Aspergillus oryzae*), is one of the most important seasoning agents in Asian cuisine [11]. Nowadays, it is popular worldwide due to its unique flavor [12]. However, growing health concerns have driven the consumers’ interest towards changing their eating lifestyle. People are turning to lower-sodium products and they express great concern over hypertension and cardiovascular diseases with excessive salt intake [13].

Table salt is considered the oldest flavoring ingredient and it is essential for human health. However, approximately 90% of sodium is consumed in the form of table salt. Sodium reduction is one of the toughest public health and food industry challenges worldwide. Many researchers have evaluated the reduction of sodium use with different methods, including salt substitutes, flavor enhancers, and structural modifications of salt particles [14,15]. Multisensory cues that improve perceived saltiness without increasing sodium content have also been considered. Recent studies support that visual cues like color intensity can change saltiness perception. For example, Sukkwai et al. [16] reported that the different levels of orange color can cause different saltiness perceptions of mayonnaise-based dipping sauces. The research indicates that color may act as a saltiness booster. Similar findings have been reported for soups, dressings, and other savory foods [17,18].

Building on the evidence that visual cues, particularly color intensity, can act as a saltiness booster in savory foods, understanding how visual color cues influence consumers’ sensory expectations and emotional responses becomes increasingly important for sodium reduction strategies. While reducing sodium content is essential for public health, it often leads to diminished saltiness perception and lower consumer acceptance, posing a major challenge for product reformulation. From a multisensory perspective, color serves as a salient pre-consumption cue that can shape expected saltiness and subsequently modulate perceived saltiness, liking, and emotional responses during consumption.

Although previous studies have demonstrated the influence of food color on saltiness perception and consumer emotion, most have focused on specific product categories such as sauces, soups, or dairy-based foods, with limited attention given to savory condiments that are major contributors to dietary sodium intake, such as soy sauce [17]. Moreover, research simultaneously examining the interaction among visual color cues, saltiness expectation, perceived saltiness, sensory liking, and emotional responses remains scarce. This gap is particularly relevant to product reformulation, where color intensity is a prominent quality attribute and saltiness often functions as a contextual and auxiliary flavor rather than a dominant taste. Addressing this gap is therefore essential for understanding how visual design strategies can be leveraged to modulate saltiness-related sensory experiences and support the development of low-sodium soy sauce products without compromising consumer satisfaction.

The objectives of this study were to investigate the effects of different color intensities on saltiness perception, expectation, sensory liking, and emotional responses for soy sauce. The results gave useful information for developing foods with low sodium content, optimal color, and high consumer satisfaction.

## 2. Materials and Methods

### 2.1. Material

Premium light soy sauce (Megachef, Marine Resources Development Co., Ltd., Bangkok, Thailand), table salt (Morton^®^ Salt, Inc., Chicago, IL, USA), and a mixed brown color solution (MBCS; Best Odor Co., Ltd., Bangkok, Thailand) composed of black, yellow, and red food-grade powders were used in the study. Commercially available soy sauces, including naturally fermented (KIKKOMAN^®^, Kikkoman Foods, Inc., Walworth, WI, USA) and chemically hydrolyzed (La Choy, ConAgra Foods^®^, Omaha, NE, USA) products, were used for reference color comparison.

### 2.2. Soy Sauce Preparation

**Figure 1:**
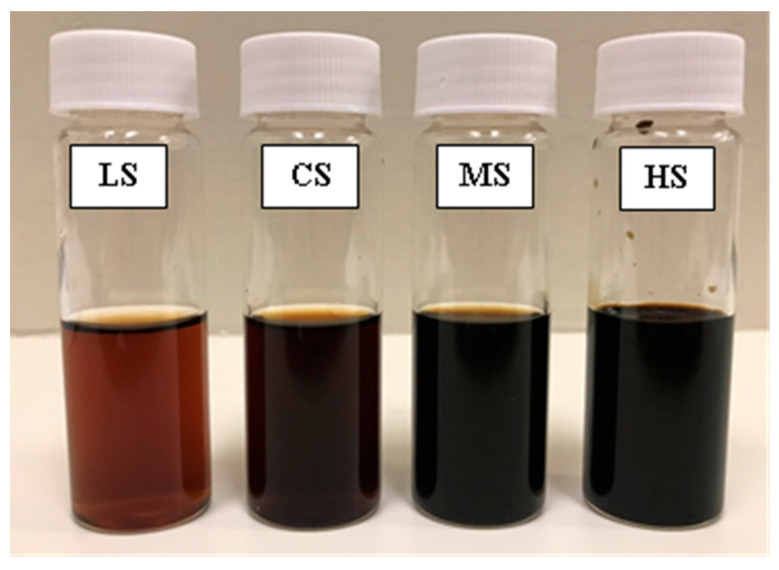
Soy sauce samples with different color intensities: LS = light, CS = control, MS = moderate, and HS = high color intensity.

Four soy sauce samples with different color intensities were prepared (Figure 1). The light-colored soy sauce (LS) was by diluting the control soy sauce to 50% concentration with distilled water. The control soy sauce (CS) was used without modification. The moderate-colored soy sauce (MS) was prepared by adding 5% (*v*/*v*) of the mixed brown color solution (MBCS) to the control sample. Lastly, the high-colored soy sauce (HS) was formulated by adding 10% (*v*/*v*) MBCS. All samples were adjusted to contain an identical salt concentration of 12% NaCl (*w*/*v*) to eliminate the influence of sodium content on sensory perception. Color measurements (CIE, L*, a*, b*) were performed in triplicate using a spectrophotometer (CM–5, Konica, Jakarta Raya, Indonesia). The total color difference (ΔE) was then calculated using the control sample as the reference, according to Poonnakasem et al. [19].

### 2.3. Consumer Expectation, Perception, Liking, Emotion and Expected JAR Color and Saltiness Intensity

This work is a collaboration between Khon Kaen University and Louisiana State University, Agricultural Center. The research procedure was in accordance with the ethics approval HE611232 by the Khon Kaen University Ethics Committee for Human Research (KKUEC), Thailand, and by the Louisiana State University Agricultural Center Institutional Review Board (IRBAG-21-0063; Baton Rouge, LA, USA).

A total of 100 participants (students, staff, and volunteers; aged = 18–50 years) who reported familiarity with soy sauce were recruited. Eligibility was based on self-reported normal color vision and taste perception, with participants confirming the absence of any known color vision or taste-related abnormalities. This self-report-based screening approach is commonly used in consumer sensory studies. Sensory evaluation was carried out in individual booths under standardized white lighting conditions.

The questionnaire, created in Qualtrics software (Qualtrics, Provo, UT, USA), was adapted from Sukkwai [20]. Each participant evaluated four coded samples (three-digit random numbers) in a randomized order.

Participants first rated their expectation of brown color and saltiness intensity based on appearance only, using a 9-point expectation scale (1 = extremely less than expected; 5 = as expected; 9 = extremely more than expected). They then rated color liking on a 9-point hedonic scale (1 = dislike extremely; 5 = neither like nor dislike; 9 = like extremely), and expected saltiness intensity using a 3-point Just-About-Right (JAR) scale (1 = not salty enough; 2 = just about right; 3 = too salty) [21].

Participants selected applicable emotion terms from the 39 food-elicited emotion descriptors of the EsSense Profile^®^ [22]. After visual evaluation, they tasted the soy sauce (poured over a cube of boiled chicken breast, served at room temperature, 32 ± 2 °C) and repeated ratings for perceived saltiness intensity, saltiness liking, and emotional responses. Palate cleansing was performed between samples using unsalted crackers and water.

### 2.4. Statistical Analysis

Color difference (ΔE) values and consumer responses were analyzed using one-way analysis of variance (ANOVA). Mean comparisons were performed by Duncan’s multiple range test at a significance level of α = 0.05. Correspondence analysis (CA) was used to visualize relationships between samples and emotion terms (XLSTAT Premium 2019, Addinsoft, Paris, France).

Paired-sample *t*-tests compared saltiness intensity scores obtained from visual versus taste evaluation. JAR data were analyzed using the Cochran–Mantel–Haenszel (CMH), Stuart–Maxwell, and McNemar tests [19,23]. All analyses were conducted using SAS Software (v.9.4; SAS Institute Inc., Cary, NC, USA) and Microsoft Excel 2010 (Microsoft Corporation, Redmond, WA, USA).

## 3. Results and Discussion

### 3.1. Effect of Visual Cues on Consumers’ Expectation of Soy Sauce Color and Saltiness, and Perceived Saltiness

The results summarized in Table 1 are the measured color differences (ΔE) and consumers’ evaluations of color and saltiness expectation for soy sauces of different color intensities. The ΔE values of LS, MS, and HS were 32.2, 38.11, and 59.56, respectively. These values are within the variability observed in commercial soy sauces in the U.S. market. For example, the ΔE values of the Kikkoman and La Choy brands are from 64.47 to 74.67 [24]. Because a ΔE value of approximately 2.3 corresponds to a just noticeable difference (JND) [24], consumers in this research were likely able to visually discriminate among the test samples.

Based on the visual evaluations, expectation scores for brown color and saltiness were highest for HS (5.81 and 6.03) and MS (5.69 and 5.78). Conversely, LS had the lowest scores at 2.34 and 3.30, respectively. A possible explanation is that the darker hues of the HS and MS samples made stronger impressions of authenticity and taste intensity. Thus, it led to higher expectations. In contrast, the lower levels of brown color of CS and LS soy sauce samples resulted in lower expectation scores. In addition, some consumers reported that the lighter-colored LS sample visually resembled fish sauce rather than soy sauce. In Asian culinary contexts, fish sauce is typically associated with a different saltiness profile, which may have led to lower saltiness expectations and influenced expectation-driven responses, independent of the actual salt content.

Increasing the brown color intensity generally led to higher scores of expected saltiness (*p* < 0.05; Table 1). According to Zampini et al. [25], consumers generated consistent flavor expectations based on visual cues. This supports the concept of color-induced flavor expectations. Paired-sample *t*-tests showed significant differences for comparisons between expected and perceived saltiness intensities. LS and CS samples were saltier than expected. HS had the lowest score in perceived saltiness (4.84; *p* < 0.05). The MS sample showed no significant difference (*p* > 0.05). Interestingly, although the HS sample had the highest expected saltiness intensity (6.03), it was lowest in perceived intensity (4.84; *p* < 0.05). In general, consumers may expect the high brown color of soy sauce to deliver high saltiness. However, the mismatch in HS may be a potential contrast effect or expectation disconfirmation. Too much dark color may raise expectations unrealistically high, which causes perceived intensity to be judged lower after tasting [7]. It was also reported by Sukkwai et al. [16]. The study found that the consumers expected the darker orange color to be saltier than the lighter orange color of mayonnaise-based dipping sauces. However, perceived saltiness decreased when color intensity was too much.

On the other hand, the MS sample achieved a balance between visual expectation and actual perception. Its moderate hue was enough dark to convey saltiness but not so intense to make over-expectation. This may explain why MS also received the highest liking scores in both color and saltiness evaluations (Table 2). Such alignment between expectation and perception is important for consumer satisfaction. It is because sensory-cognitive harmony generally improves hedonic experience [26].

Previous studies indicate that sensory expectations are shaped by prior experiences and familiarity with similar products [27,28]. These expectations can also influence product acceptance and willingness to pay [29]. Apaolaza et al. [30] described this phenomenon as the halo effect. It is the evaluation of one sensory attribute biases judgments of others. Similar halo or assimilation effects have been reported in cross-modal food perception with color, taste, and aroma [31]. In general, these findings suggest that color not only impacts expectation but also modulates post-consumption perception and emotional responses.

### 3.2. Liking and JAR Responses for Expected and Perceived Saltiness, and Emotion of Soy Sauce

Table 2 is the mean liking scores for color and saltiness in the four soy sauce samples. Brown color liking was highest for HS and MS (5.85 and 6.17, respectively), followed by CS (5.39). LS (3.98) had the lowest score (*p* < 0.05). A similar trend was seen in saltiness liking. MS (6.30) and HS (5.93) scored higher than CS (5.86) and LS (5.78). These results suggest that consumers generally preferred samples with darker brown coloration over lighter ones.

The visual appearance of soy sauce greatly contributed to perceived quality and flavor intensity. This is because color is often used as a heuristic cue for flavor strength and product authenticity [32]. Jimenez et al. [32] noted that visual cues often cause anticipatory evaluations. Consumers typically imagine how good a food will taste before consumption, which creates their hedonic expectations.

The highest saltiness-liking score for MS (Table 2) corresponded well with its expected saltiness intensity (Table 1). It suggests that moderate color intensity made a consistency between expectation and perception. This harmony often results in higher satisfaction. Consumers consider the product as “taste as it looks” [33]. In contrast, despite HS having a darker hue, it had slightly lower liking scores. It could possibly be an over-expectation effect that too much dark color may cause saltiness expectations that are not confirmed upon tasting [34]. Darker coloration improved initial expectations of flavor but did not always improve actual liking. Product appearance impacts consumer acceptance, but it may not be the biggest driver of overall preference. Other sensory modalities like taste, aroma, and texture are also the main drivers [7,35,36].

Pairwise comparisons of saltiness intensity based on visual and taste evaluations are in Table 3. Most visual sample pairs had significant differences, especially between LS and the darker samples (MS and HS). Consumers clearly distinguished saltiness expectations by color intensity. In contrast, differences for sample pairs during tasting were mostly nonsignificant. Thus, actual saltiness perception was more uniform once the samples were tasted.

**Figure 2:**
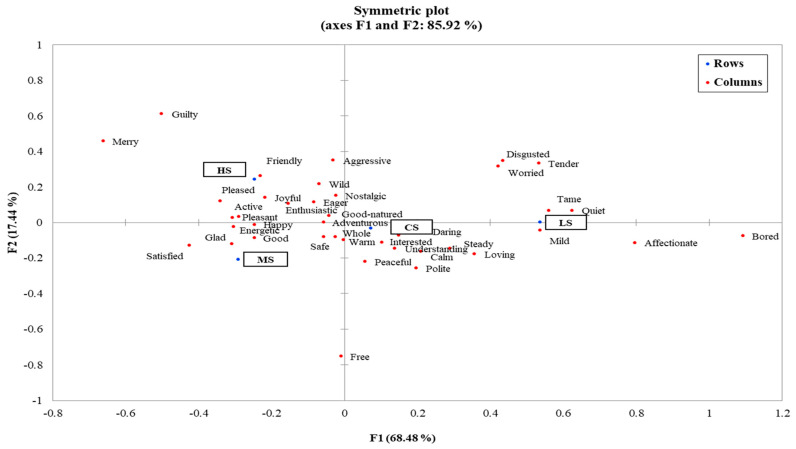
Correspondence analysis (CA) plot between difference colorant of soy sauce and emotional responses (Visual testing): See Figure 1 for sample descriptions.

**Figure 3:**
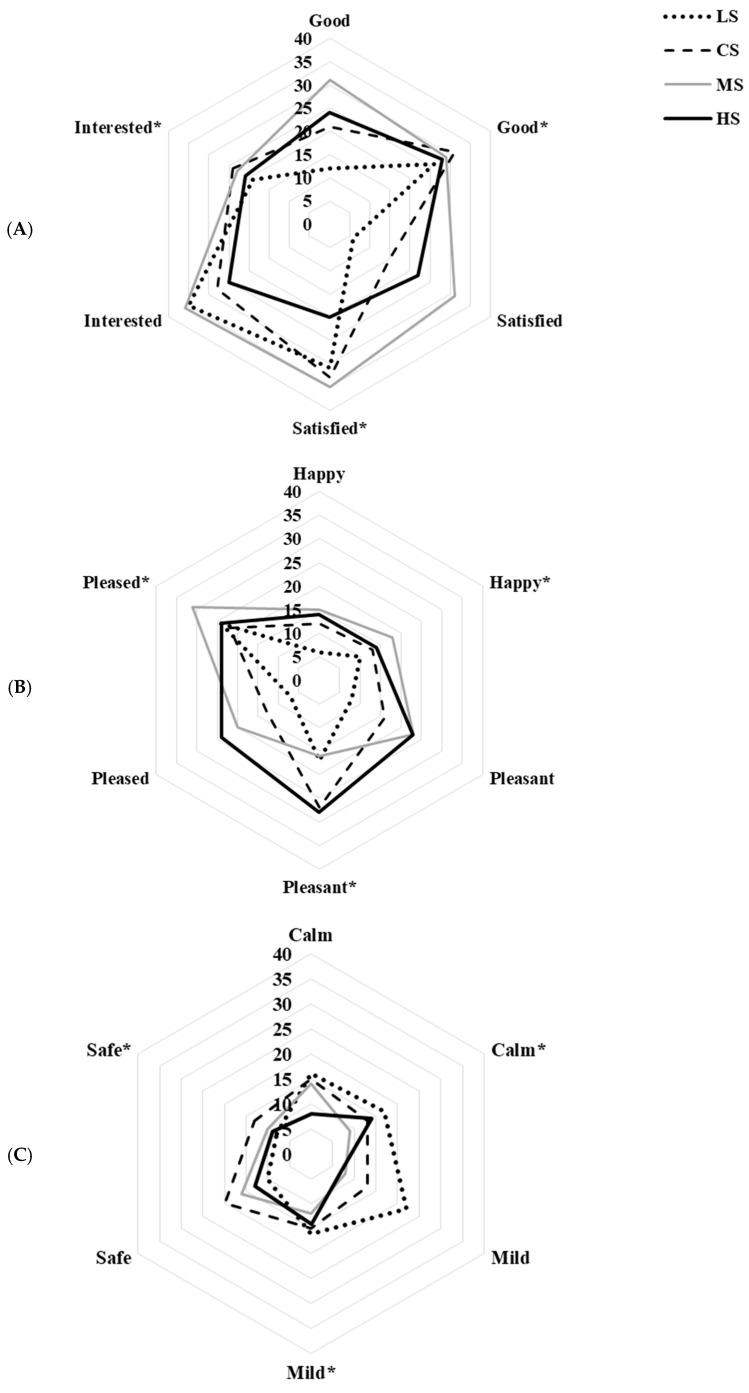
Emotional frequencies of soy sauce visual testing vs. taste testing (*): (**A**) for good, satisfied and interested, (**B**) for happy, pleased, and pleasant and (**C**) for calm, safe, and mild. Based on 100 consumer responses.

**Figure 4:**
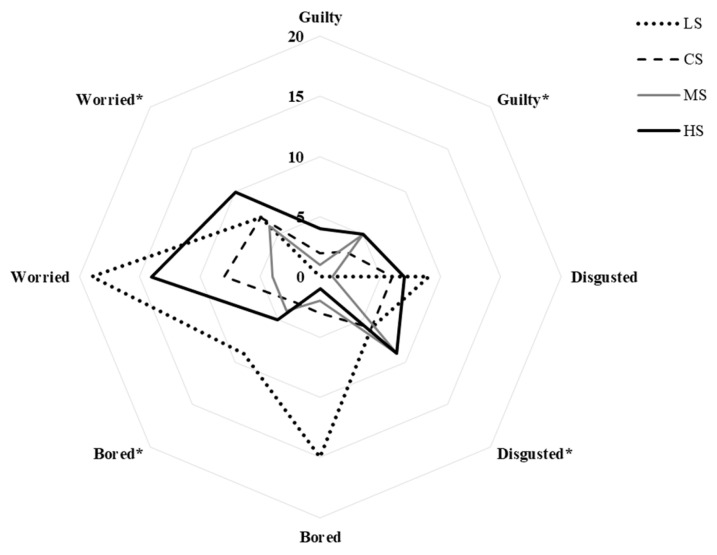
The frequencies of negative emotion of soy sauce visual testing vs. taste testing (*): guilty, disgusted, bored, and worried emotion terms. Based on 100 consumer responses.

Analysis of JAR responses was conducted to compare visually evoked and perceived saltiness intensities. The results show that there was an incongruence between expectation and actual taste in LS, CS, and HS samples (Table 4). This means that visual cues alone did not correctly predict saltiness perception. Only MS samples had cross-modal congruence. Their color-induced expectations aligned with the actual taste experience. Category responses for “too salty” were highest for HS (45%) in visual evaluation, which confirms that darker hues can increase saltiness expectations. These findings support the view that consumers integrate visual and gustatory information through cross-modal correspondence, but excessive visual stimulation may disrupt this integration [37,38,39]. In addition, emotional responses for soy sauce samples were further examined using correspondence analysis (CA) (Figure 2). Both dimensions F1 and F2 explain 85.92% of the total variance. The CA biplot showed that LS was associated with negative emotion terms (disgusted, bored, worried). Meanwhile CS, MS, and HS had associations with positive emotions. The frequency distributions of positive and negative emotion terms are in Figure 3 and Figure 4, respectively. Under visual evaluation, MS had the highest frequencies for interested (36%), satisfied (31%), good (31%), and happy (18%). For HS, the main emotions were pleased (24%) and pleasant (23%). LS was calm (16%) and mild (22%). The safe emotion term (20%) was most frequent for CS. These results are similar to earlier studies. It shows that positive emotions such as happy, satisfied, and pleasant are commonly associated with foods that meet sensory expectations and have perceived quality [40].

Visual versus taste evaluations were compared. The frequency of interested and safe emotions decreased via treatments after tasting (Figure 3A,C). Conversely, satisfied, pleased, happy, good, and pleasant increased (Figure 3A,B). This suggests that actual consumption improved positive affective responses. This pattern was most pronounced for the MS sample, where tasting enhanced satisfaction and pleasure, reinforcing its hedonic superiority. Similar results were presented by Sukkwai et al. [16]. They found that no colorant and moderate colorant of mayonnaise-based dipping sauces had higher scores for good, interested, and satisfied emotions than high-colored samples. These results support the notion that balanced visual cues can help both sensory and emotional appeal rather than too much or too little coloration [41].

Regarding color-evoked negative emotion terms (Figure 4), the LS soy sauce sample had the highest frequencies for disgusted (9%), bored (15%), and worried (19%). Thus, too much pale color can make negative reactions. Moreover, both LS and HS soy sauce samples triggered more negative emotions than CS and MS samples. It indicates that either too light or too dark tends to reduce sensory appeal. However, negative emotion terms decreased when the consumers tasted the samples. There were more positive emotions. Thus, direct sensory experience can reduce color-induced bias. For negative emotions, guilty showed minimal variation throughout treatments.

Overall, moderate brown color (MS) offered a good balance between expectation, liking, and emotion. This result supports that a visual stability between product appearance and taste intensity boosts both hedonic liking and positive emotions. Food-elicited emotions are known to come from a complex interplay of sensory cues, past experiences, and cultural or personal links [42]. However, soy sauce functions within a complex application system, where its saltiness typically acts as an auxiliary (rather than dominant) flavor and is susceptible to environmental influences. To address this, our study adopted a ‘soy sauce–meat (chicken)’ model, using chicken to create a standardized flavor background. This enabled a focused examination of soy sauce’s auxiliary or relative saltiness within this composite flavor system. From the perspective of expectation–disconfirmation theory, the incongruence between color-induced saltiness expectations and actual taste perception may result in negative disconfirmation, leading to cognitive dissonance and influencing both hedonic and emotional responses. Finally, within Asian culinary traditions, the characteristic brown hue of soy sauce has become tightly linked to its core salty taste through long-term cultural exposure. This perceptual association forms a critical premise of our study and suggests that its findings and applications may be particularly relevant to Asian populations. Their generalizability to other cultural or dietary contexts requires further investigation.

These findings provide a deeper understanding of how cross-modal congruence and visual design can support healthier and low-sodium foods without reducing consumer pleasure.

## 4. Conclusions

Increasing the brown coloring of soy sauce influenced the visual expectation of saltiness, color liking, saltiness liking, and emotional responses. When comparing the visual responses against the taste responses, the dark brown colored soy sauce (HS) showed incongruence between the color-evoked expectations and perceived saltiness intensity at 12% NaCl. In contrast, reducing the color concentration of soy sauce made negative emotions such as disgusted, bored, and worried, while increasing the brown coloring of soy sauce can increase positive feelings like interested, satisfied, and pleasant. Overall, the results demonstrate that changing the brown color intensity of soy sauce can effectively control saltiness expectation and perception. It is also associated with positive and negative emotional responses. However, the human data in this study are primarily based on self-reports and, therefore, the findings warrant further validation. Given the current lack of objective methods directly comparable to self-reports, this study employed multiple established sensory evaluation techniques to achieve cross-validation, thereby enhancing the reliability and robustness of the results.

### 4.1. Practical Implications

The results suggest that adjusting the brown color intensity of soy sauce can serve as a practical, non-invasive strategy to support sodium reduction by shaping saltiness expectations and emotional responses without reducing consumer acceptance. Such visual design approaches may complement conventional reformulation methods and aid the development of healthier, low-sodium products, particularly in savory condiments.

### 4.2. Limitation

The findings of this study should be interpreted within the context of the experimental design. Sensory and emotional responses were primarily assessed using self-reported measures. Color intensities were achieved through dilution, which may have affected other sensory attributes beyond saltiness; however, the color intensity of all samples was objectively measured to ensure consistency across conditions. In addition, soy sauce was evaluated within a food matrix, and the results may be influenced by cultural familiarity with soy sauce.

## Figures and Tables

**Table 1:** ΔE value and expectation of brown color intensity and saltiness intensity based on visual testing vs. taste testing of soy sauces.

Samples	Expectation Based on Visual Testing ^1^		Expectation Based on Taste Testing ^1^	Color Measurement ^+^
	Brown Color Intensity	Saltiness Intensity	Saltiness Intensity	ΔE
**LS**	2.34 ± 1.75 ^c^	3.30 ± 1.96 ^c^	5.56 ± 2.05 ^a,^*	32.20 ± 0.53 ^c^
**CS**	4.46 ± 1.26 ^b^	4.61 ± 1.24 ^b^	5.66 ± 1.62 ^a,^*	0.00 ± 0.00 ^d^
**MS**	5.69 ± 1.30 ^a^	5.78 ± 1.45 ^a^	5.46 ± 1.64 ^a^	38.11 ± 0.20 ^b^
**HS**	5.81 ± 1.43 ^a^	6.03 ± 1.40 ^a^	4.84 ± 1.81 ^b,^*	59.56 ± 0.56 ^a^

^1^ Mean ± SD from 100 consumer responses based on a 9-point expectation scale: ^+^ Mean ± SD from 3 replication: * indicates significant difference the paired *t*-test between saltiness intensity based on visual and taste testing (*p* < 0.05): Difference letters in the same column indicate significant difference by Duncan’s multiple range test (*p* < 0.05): See Figure 1 for sample descriptions.

**Table 2:** Color liking and saltiness liking score of different colorant soy sauces.

Samples	Color Liking ^1^	Saltiness Liking ^1^
LS	3.98 ± 1.85 ^c^	5.78 ± 1.96 ^b^
CS	5.39 ± 1.59 ^b^	5.86 ± 1.82 ^ab^
MS	6.17 ± 1.59 ^a^	6.30 ± 1.80 ^a^
HS	5.85 ± 1.63 ^ab^	5.93 ± 1.88 ^ab^

^1^ Mean ± SD from 100 consumer responses based on a 9-point hedonic scale: Difference letters in the same column indicate significant difference by Duncan’s multiple range test (*p* < 0.05): See Figure 1 for sample descriptions.

**Table 3:** Pairwise comparison of saltiness intensity based on visual and taste of sample pairs using the McNemar test ^a^.

Sample Pairs	*χ*2 Value for Expectation of Saltiness Intensity Based on Seeing	*χ*2 Value for Saltiness Intensity Based on Tasting
**LS/CS**	23.13	6.50
**LS/MS**	48.34	10.45
**LS/HS**	62.01	2.06 ^b^
**CS/MS**	19.11	0.76 ^b^
**CS/HS**	30.25	0.84 ^b^
**MS/HS**	0.90 ^b^	3.68 ^b^

^a^ The critical *χ*2 value = 5.99 at *df* = 2 and *α* = 0.05: ^b^ No significant difference between samples: See Figure 1 for sample descriptions.

**Table 4:** Saltiness perception based on the JAR responses as indicated by color and taste.

Samples	Modes	Saltiness Intensity (%)			Congruence with CMH *
		Not Salty Enough	JAR	Too Salty	
LS	Color	68	17	15	<0.0001
	Taste	28	51	21	
CS	Color	38	45	17	<0.0001
	Taste	14	53	33	
MS	Color	8	52	40	0.2963
	Taste	9	59	32	
HS	Color	4	51	45	0.003
	Taste	19	54	27	

* Significant difference in the JAR score across products (*p* < 0.05): See Figure 1 for sample descriptions.

## Data Availability

The original contributions presented in the study are included in the article. Further inquiries can be directed to the corresponding author.
